# Post‐Hospital Access to Preferred and High‐Quality Skilled Nursing Facilities for Patients With Opioid Use Disorder

**DOI:** 10.1111/1475-6773.70110

**Published:** 2026-03-31

**Authors:** Talia S. Benheim, Momotazur Rahman, Andrew R. Zullo, Ashley Z. Ritter, Simeon D. Kimmel, Patience M. Dow

**Affiliations:** ^1^ Department of Health Services, Policy, and Practice Brown University School of Public Health Providence Rhode Island USA; ^2^ Department of Epidemiology Brown University School of Public Health Providence Rhode Island USA; ^3^ Hunter‐Bellevue School of Nursing, Hunter College, City University of New York New York New York USA; ^4^ Section of General Internal Medicine, Boston University Chobanian and Avedisian School of Medicine and Boston Medical Center Boston Massachusetts USA; ^5^ Section of Infectious Diseases, Boston University Chobanian and Avedisian School of Medicine and Boston Medical Center Boston Massachusetts USA

**Keywords:** care transitions, Medicare, opioid use disorder, post‐acute care, skilled nursing facility

## Abstract

**Objective:**

To examine whether Medicare beneficiaries with opioid use disorder (OUD) encounter limited access to hospitals' highest‐volume (i.e., “preferred”) or high‐quality skilled nursing facilities (SNFs) compared to beneficiaries without OUD.

**Study Setting and Design:**

We estimated within‐hospital disparities in access to preferred and high‐quality SNFs by OUD status using linear probability models and discrete choice models (McFadden‐style conditional logistic regression). We defined preferred status using shared hospital‐SNF discharge volume and quality using CMS star ratings. In choice models, we matched patients with and without OUD 1:1 on discharging hospital and date, and applied inverse probability weighting and propensity score subclassification to address confounding.

**Data Sources and Analytic Sample:**

We used 2017–2021 Medicare inpatient claims to identify Medicare beneficiaries ages 18+ discharged to a SNF following hospitalization.

**Principal Findings:**

In the full sample (*N* = 6,490,593), patients with OUD were 2.5 and 3.6 percentage points (pp) less likely to enter preferred and high‐quality SNFs, respectively. Among those discharged to preferred SNFs, patients with OUD were 2.0 pp less likely to enter high‐quality preferred SNFs. In the matched subsample (*n* = 156,610), the marginal effect of preferred status on a person being discharged to their closest SNF was 1.1 pp lower for patients with OUD than those without OUD (*p* < 0.05), but with no significant disparity after inverse probability weighting. When the closest SNF's quality rating increased by 1 star, the probability of entry increased by 0.7 pp for people without OUD but decreased by 0.2 pp for people with OUD (difference = 0.9 pp, *p* < 0.001), a difference that persisted after weighting.

**Conclusions and Relevance:**

Publicly‐reported star ratings had weaker associations with the SNF placements of Medicare beneficiaries with OUD compared to those without OUD, and preferred referral networks alone did not eliminate these gaps. Regulatory and reimbursement reforms that support SNFs in developing OUD‐related care capacity and that promote equitable admissions deserve attention.

## Callout Box

- What is known on this topic?
  ○ Patients with opioid use disorder (OUD) are frequently rejected from the skilled nursing facilities (SNFs) to which they are referred after hospital discharge.
  ○ Hospitals are increasingly developing networks of “preferred” SNFs, where they send high volumes of referrals and selectively invest in improving care coordination, communication, and information‐sharing systems.
  ○ Hospital‐SNF referral relationships and SNF star ratings are associated with improved health outcomes.
- What this study found?
  ○ Higher star ratings increased the probability of SNF placement for patients without OUD, but decreased the probability of placement for patients with OUD.
  ○ Preferred status had a weaker association with the SNF placements of patients with OUD than those without OUD, a finding that was not robust to adjusting for other patient characteristics.
  ○ Preferred SNF status did not entirely reduce disparities in the quality of SNFs where patients with versus without OUD were placed.

## 1 Introduction

Hospitalized patients with opioid use disorder (OUD) and co‐occurring skilled care needs are increasingly discharged to skilled nursing facilities (SNFs) [1], but may be less likely to enter SNFs with high‐quality ratings than those without OUD [2]. Although patients with OUD represent a relatively small proportion of the overall SNF population, they often have complex medical or behavioral health needs requiring specialized services and care [3, 4, 5, 6, 7]. SNF admission decisions, hospital referral practices, and the limited number of facilities able to meet these specialized needs may all contribute to disparities in SNF placement. Patients with OUD are disproportionately rejected by the SNFs they are referred to for post‐acute care [8, 9, 10, 11, 12], with commonly reported barriers centered around care delivery logistics (e.g., medication for OUD), staffing and training deficits, inadequate financial incentives, concerns around regulatory scrutiny, and perceived facility fit [3, 4, 12, 13, 14, 15, 16, 17, 18, 19]. Some evidence also suggests that hospitals preferentially refer patients with OUD to a smaller subset of SNFs which have higher likelihoods of accepting patients with OUD but lower average quality metrics (e.g., lower star ratings, fewer direct patient care hours per day) [9, 10]. However, there have not yet been national analyses of how hospital‐SNF referral linkages and SNF quality independently or jointly influence SNF placement disparities for patients with OUD.

Both hospital‐SNF referral relationships and SNF quality ratings can impact patient outcomes after hospital discharge, which makes them important considerations for patients with OUD [20, 21, 22, 23, 24]. Under value‐based payment systems that incentivize hospitals to reduce readmissions and shorten hospital stays, hospitals are increasingly developing networks of “preferred” SNFs. These are facilities where hospitals send high volumes of referrals and invest in improving care coordination, communication, and information‐sharing systems [20, 25, 26, 27]. These investments could benefit patients with OUD, for whom coordination failures during post‐acute care transitions have been reported to disrupt care and erode trust [3]. Discharge to SNFs with stronger hospital referral linkages is associated with lower readmission rates [20, 21, 22, 23, 24], and discharge to SNFs with high star ratings is linked to reduced readmissions, mortality, and SNF length of stay [28, 29]. Hospitals often select SNFs to include in their preferred networks based on existing high‐volume relationships and SNF quality metrics [20, 22, 30, 31, 32], though some hospitals report strategically partnering with lower‐quality SNFs to ensure quicker discharge for patients who are harder to place [33].

It is unclear whether patients with OUD would have equitable access to hospitals' preferred SNFs. On one hand, hospitals may hold their preferred SNFs accountable for admitting patients with complex care needs, such as OUD, in exchange for maintaining preferred status and continuing to receive the referrals that come with it [26, 27]. Since preferred SNFs are typically higher quality facilities [20, 22, 30, 31, 32], this could help reduce quality‐related SNF disparities for patients with OUD. On the other hand, the consistent referral flows that preferred and high‐quality SNFs receive may also give these facilities leverage to screen out patients with complex care needs or perceived as less “profitable” [26, 27, 34, 35, 36, 37, 38]. While better information sharing capacity at preferred SNFs, such as sharing of electronic records, could improve care transitions by ensuring SNFs can meet patients' care needs [3], it could also make it easier for preferred SNFs to observe patient characteristics before admission, potentially facilitating selectivity [3, 20, 25, 26]. Decreased access to preferred and high‐quality SNFs has been documented among patients with dementia and serious mental illness [26, 34, 39, 40], and it is plausible that patients with OUD face similar barriers.

We examined whether hospitalized Medicare beneficiaries with OUD have a different overall likelihood of going to hospitals' preferred or high‐quality SNFs, and whether those who enter preferred SNFs have equitable access to high‐quality preferred facilities. We then used choice models to assess the independent influences of preferred relationships and quality on SNF placement decisions for patients with versus without OUD. If individuals with OUD experience limited access to preferred or high‐quality SNFs, then clinical and policy changes may be needed to ensure equitable access to the potentially improved care transitions and outcomes that occur at these SNFs.

## 2 Methods

### 2.1 Data Sources

We identified hospital discharges using inpatient claims from the 100% Medicare Provider Analysis and Review (MedPAR) files and SNF admissions using the Residential History File algorithm [41]. We obtained information on beneficiary demographics and location from the Master Beneficiary Summary File; SNF characteristics, star ratings, and location from the Certification and Survey Provider Enhanced Reports (CASPER) and Nursing Home Compare datasets; and hospital location from the American Hospital Association Annual Survey.

### 2.2 Study Cohort

We included hospital discharges of Medicare beneficiaries ages 18+ who were admitted to a SNF within 1 day of discharge between 2017 and 2021 in the 50 U.S. states or D.C. We excluded discharges if the patient had any SNF stay in the year prior to the hospitalization because the setting of a prior SNF stay could systematically influence subsequent SNF placement. We excluded discharges to SNFs located in a different state than the discharging hospital (2.4%) to ensure that all SNFs within each individual's choice set share the same regulatory environment, which supports the conditional logit model's independence assumption. We also excluded discharges that were missing relevant hospital, SNF, or geocoding information. See Figure S1 for the sample selection flowchart.

### 2.3 Exposure

The primary exposure was OUD status defined by the presence of an ICD‐10 diagnosis code for OUD, opioid dependence, or opioid overdose, or procedure codes for OUD treatment in any position on the inpatient claim directly preceding the SNF admission (Table S1).

### 2.4 SNF Preferred Status

One outcome of interest was whether the patient's chosen SNF was a “preferred” SNF of the discharging hospital, conditional on the full set of SNFs to which they could be discharged. We use the term “choice” to describe the SNF the patient was admitted to for consistency with the discrete choice model literature [39, 42, 43, 44]. This term is not intended to imply that patients independently selected their SNF, as SNF placement involves multiple decision‐makers and factors outside a patient's control, such as bed availability and insurance coverage [34, 45].

We defined choice sets at the hospital‐year level under the assumption that patients discharged from a given hospital in a certain year choose from the same set of SNFs. In alignment with previous studies [39, 43, 44], each hospital‐year choice set included SNFs in any of the following categories: (a) all SNFs to which at least one patient from the hospital was discharged, (b) all SNFs within 22 km (13.7 miles) of the discharging hospital, and (c) the 15 SNFs closest to the hospital. This approach prevents the choice sets from being too restrictive for hospitals in rural areas, where potential SNFs may be further than 22 km away, nor too restrictive in urban settings, where hospitals have more than 15 nearby SNFs, and it ensures that each patient's selected SNF is within their choice set. The 22 km threshold corresponds to approximately the 80th percentile of patient travel distances to their selected SNF. We computed geodetic distances using *geodist* in Stata [46]. We used exact latitudes and longitudes to compute distances between the discharging hospital and SNF. As in prior studies [26, 34, 35], we rank‐sorted each SNF in the hospital's choice set by shared discharge volume and classified the highest volume SNFs that received a cumulative 50% of a hospital's prior year's discharges as “preferred.” In a sensitivity analysis, we tested whether our findings were robust to alternative thresholds of cumulative discharges used to define “preferred” SNFs (10%, 20%, 30%, 40%, 60%, 70%, and 80%).

### 2.5 SNF Quality

A second outcome of interest was SNF quality, measured using 5‐star ratings from the Nursing Home Compare datasets from the month of hospital discharge. We used the overall rating, which is a composite of ratings on staffing, health inspections, and quality measures.

### 2.6 Analysis

Our analyses included: (1) linear probability models to estimate differences in the probability of entering preferred or high‐quality (4–5 star) SNFs, and (2) discrete choice models, which more closely reflect the real‐world SNF selection process by simultaneously accounting for the attributes of the full set of SNFs available at discharge.

### 2.7 Linear Probability Models

We ran linear probability models with hospital fixed effects to examine the within‐hospital associations between OUD and the probability of entering a preferred or high‐quality SNF. Models were adjusted for age, race/ethnicity (as recorded), sex (as recorded), dual‐eligible status, Rural–Urban Commuting Area (RUCA) code of home zip code, and modified Elixhauser index (excluding alcohol and substance use disorder codes).

### 2.8 Choice Models

We used McFadden‐style conditional logit models to estimate the probability of entering a specific SNF based on the observable attributes of all competing SNFs available to a patient at discharge. Because conditional logit models include individual fixed effects, patient‐level covariates that do not vary across SNFs in the individual's choice set cannot be directly entered into the models. Therefore, to address patient‐level confounding between patients with and without OUD, we first 1:1 matched each OUD hospitalization with a non‐OUD hospitalization on the discharging hospital and date (+/−2 days) to ensure comparisons were made among patients with a common choice set of SNFs and similar time‐dependent discharge conditions. In sensitivity analyses, we tested robustness to different matching ratios (using up to 2 and up to 3 non‐OUD matches per OUD hospitalization). We then applied two propensity score‐based methods. First, we used inverse probability of treatment weights (IPW) based on propensity scores. Propensity scores were estimated using a logit model with OUD as the dependent variable and the following covariates: age (modeled as a two‐piece linear spline), race/ethnicity (as recorded), sex (as recorded), dual‐eligible status, Medicare advantage enrollment, Medicare eligibility due to end‐stage renal disease, RUCA code of home zip code, and modified Elixhauser comorbidity index. We applied IPW to the conditional logit model to obtain overall estimates adjusted for patient‐level differences. Second, we stratified the matched cohort into propensity score quintiles and re‐estimated the conditional logit model within each quintile to examine whether effects varied across patients with different likelihoods of having OUD. We provide descriptive statistics for the full, 1:1 matched, and IPW‐weighted samples to show how these steps affect the sample composition. We also descriptively plotted, within the 1:1 matched sample, the proportion discharged to preferred and high‐quality SNFs by propensity score quintile, as well as the share entering high‐quality preferred SNFs among preferred SNF discharges.

To carry out the conditional logit model, within the matched subsample, we constructed a dataset with as many observations per individual as there were SNFs in the individual's choice set, with a dependent variable, “choice,” that equaled 1 if the individual entered that SNF and 0 otherwise. The model estimates the relative importance of SNF characteristics on the probability that a patient “chooses” a given SNF. We included the following SNF characteristics in the model: preferred status, quality, distance to home zip code centroid, distance to discharging hospital, ownership, multifacility/chain affiliation, hospital‐affiliation, bed size, occupancy rate, and percentage of residents with Medicaid. To test whether SNF characteristics influenced “choice” differently for patients with versus without OUD, we included interaction terms between OUD and each SNF characteristic. The interaction terms for preferred status and quality were our pre‐specified coefficients of interest. We clustered standard errors by state.

### 2.9 Marginal Effect Estimation

In conditional logit models, marginal effects differ for each alternative in each choice set. Therefore, we estimated marginal effects specific to the closest SNF (to the home zip code) in each person's choice set. We conducted counterfactual simulations that manipulated the preferred status or quality star rating of each patient's closest SNF. We then calculated the change in predicted probabilities of entering the closest SNF when it is preferred versus non‐preferred, or when quality increases by 1 star, holding other characteristics of the SNF and alternative SNFs fixed. We compared the average changes in predicted probabilities separately by OUD diagnosis.

All conditional logit specifications assume independence of irrelevant alternatives (IIA), meaning that the relative probability of choosing one SNF over another should be unaffected by adding or removing unrelated options. In a sensitivity analysis, we re‐estimated models after randomly removing 5%–20% of non‐chosen SNFs from each choice set to assess coefficient stability under different choice set compositions.

Data were analyzed using Stata version 18. Two‐sided *p* values < 0.05 were considered statistically significant. The Brown University Institutional Review Board approved this study. Additional details about the data and code can be found in the Brown Digital Repository (https://doi.org/10.26300/wqhr‐gz06).

## 3 Results

The full sample included 6,490,593 discharges, of which 80,439 (1.2%) had evidence of OUD. For the vast majority of SNFs, patients with OUD were a small portion of their total Medicare admissions from hospitals over the study period. About 22% of SNFs had no admissions of patients with OUD, while the median SNF had an OUD case volume of 0.8%, the 75th percentile was 1.7%, and the 99th percentile was 8.1% (Figure S3; Table S2). In the 1:1 matched subsample (*n* = 156,610), patients with OUD were, on average, nearly a decade younger (mean = 69.8 vs. 78.8 years) and more likely to be dually‐eligible for Medicaid (42.8% vs. 24.2%). Differences were substantially reduced after applying IPW (Table 1; Figure S2). In the matched subsample, patients with OUD were more likely to enter SNFs that were for‐profit (78.3% vs. 69.9%) or part of a chain (65.3% vs. 60.6%), and less likely to go to SNFs that were hospital‐affiliated (3.6% vs. 4.6%). SNFs of patients with OUD had higher percentages of Medicaid‐funded residents (mean = 52.0% vs. 47.8%) and lower star ratings (mean = 3.4 vs. 3.6). These differences remained significant after adjusting for patient‐level characteristics (Table 2).

**TABLE 1:** Patient‐level characteristics before and after matching/weighting, by opioid use disorder diagnosis.

Characteristic	Full sample		1:1 matched sample^a^		1:1 matched sample with IPW^b^	
	*N* = 6,490,593		*n* = 156,610		*n* = 156,610	
	Non‐OUD	OUD	Non‐OUD	OUD	Non‐OUD	OUD
	(*n* = 6,410,154)	(*n* = 80,439)	(*n* = 78,318)	(*n* = 78,292)	(*n* = 78,318)	(*n* = 78,292)
Age, mean (SD)	79.0 (10.6)	69.9 (11.5)	78.8 (10.7)	69.8 (11.5)	74.2 (12.2)	74.5 (12.2)
18–34 years^c^	0.1%	0.6%	0.1%	0.5%	0.5%	0.3%
35–49 years^c^	0.8%	4.0%	0.9%	4.0%	2.9%	2.4%
50–64 years^c^	6.7%	23.2%	6.8%	23.2%	13.9%	15.6%
65–79 years^c^	41.1%	52.4%	42.1%	52.5%	47.9%	46.2%
80+ years^c^	51.4%	19.8%	50.2%	19.8%	34.8%	35.6%
Female	60.3%	59.3%	59.9%	59.3%	60.0%	60.6%
Race/ethnicity						
Unknown	0.6%	0.6%	0.6%	0.6%	0.6%	0.6%
Non‐Hispanic White	79.7%	80.4%	79.2%	80.3%	79.7%	80.1%
Black or African American	10.8%	10.9%	10.4%	11.0%	10.8%	10.4%
Other	0.6%	0.5%	0.6%	0.5%	0.6%	0.6%
Asian/Pacific Islander	2.0%	0.7%	2.1%	0.8%	1.4%	1.5%
Hispanic	5.9%	6.1%	6.4%	6.1%	6.2%	6.1%
American Indian/Alaska Native	0.4%	0.7%	0.6%	0.7%	0.7%	0.6%
Urbanicity						
Metropolitan	83.2%	85.3%	85.9%	86.2%	85.8%	85.8%
Micropolitan	9.8%	9.0%	8.6%	8.5%	8.8%	8.8%
Small town	4.2%	3.3%	3.2%	3.0%	3.1%	3.2%
Rural	2.8%	2.4%	2.3%	2.2%	2.3%	2.3%
Dual‐eligible	24.2%	42.8%	25.1%	42.7%	34.5%	33.6%
Enrolled in Medicare advantage	36.9%	39.1%	38.4%	39.4%	38.9%	38.9%
Eligibility due to ESRD	2.6%	2.6%	2.5%	2.6%	2.5%	2.5%
Modified Elixhauser index	5.0 (2.5)	5.3 (2.4)	5.0 (2.5)	5.3 (2.4)	5.2 (2.5)	5.2 (2.4)
Length of stay (days), mean (SD)^c^	7.6 (7.6)	9.3 (9.3)	7.7 (7.8)	9.3 (9.3)	8.3 (8.7)	8.6 (8.5)
Hospital‐SNF distance (km), median (IQR)^c^	7.4 (2.8–17.0)	7.3 (2.7–16.5)	7.3 (2.8–16.4)	7.3 (2.8–16.4)	7.5 (2.9–16.9)	7.1 (2.7–15.7)
Home‐SNF distance (km), median (IQR) ^c^	8.3 (3.7–17.8)	9.1 (4.1–19.6)	8.3 (3.7–17.9)	9.1 (4.1–19.4)	8.6 (3.9–18.6)	8.7 (3.9–18.5)

Abbreviations: ESRD, end‐stage renal disease; IPW, inverse probability weighting; IQR, interquartile range; km, kilometers; OUD, opioid use disorder; SD, standard deviation; SNF, skilled nursing facility.

^a^ Matched 1:1 on discharging hospital and date of discharge (+/−2 days).

^b^ Estimated with inverse probability weighting based on propensity scores within matched sample. Propensity scores were estimated as a function of age, sex, race and ethnicity, urbanicity, dual‐eligible status, Medicare advantage, eligibility due to ESRD, and modified Elixhauser index.

^c^ Variable not directly used for matching or weighting.

**TABLE 2:** Characteristics of skilled nursing facilities in full population and matched subsample, by opioid use disorder diagnosis.

SNF characteristic	Non‐OUD	OUD	Unadjusted absolute difference (OUD minus non‐OUD)	Adjusted absolute difference (OUD minus non‐OUD)
Full population (*N* = 6,490,593)				
Ownership				
For‐profit	69.9%	78.3%	8.3***	2.0***
Non‐profit	26.8%	19.2%	−7.7***	−2.0***
Government	3.2%	2.5%	−0.7***	−0.0
Multifacility chain	60.6%	65.3%	4.7***	0.4*
% Occupancy rate, mean (SD)	80.7 (15.0)	80.3 (14.7)	−0.4***	0.1
Bed size, mean (SD)	129.8 (77.1)	129.6 (74.9)	−0.2	1.0
Hospital‐affiliated	4.6%	3.6%	−1.0***	−0.5***
% Medicaid, mean (SD)	47.7 (25.7)	52.1 (25.6)	4.4***	1.9***
Overall quality star rating, mean (SD)	3.6 (1.3)	3.4 (1.4)	−0.2***	−0.1***
Matched subsample (*n* = 156,610)				
Ownership				
For‐profit	72.9	78.3	5.4***	2.0***
Non‐profit	24.4	19.2	−5.2***	−1.9***
Government	2.7	2.5	−0.2*	−0.1
Multifacility chain	63.1	65.4	2.2***	0.8**
% Occupancy rate, mean (SD)	80.4 (14.9)	80.4 (14.7)	−0.0	0.1
Bed size, mean (SD)	127.3 (75.2)	130.0 (75.8)	2.8***	0.8
Hospital‐affiliated	4.1	3.6	−0.5***	−0.4***
% Medicaid, mean (SD)	47.7 (25.8)	51.9 (25.6)	4.2***	1.4***
Overall quality star rating, mean (SD)	3.6 (1.3)	3.4 (1.4)	−0.2***	−0.1***

*Note:* ***p < 0.001, **p < 0.01, *p < 0.05. The matched subsample was matched 1:1 on discharging hospital and date of discharge (+/−2 days). The means and percentages in this table are not adjusted with inverse probability weights. Absolute differences are calculated using *t*‐tests on the equality of means. Adjusted differences are calculated based on linear regression/linear probability models with hospital fixed effects. The outcomes are SNF characteristics and the models are adjusted for patient‐level characteristics, including age, sex, race and ethnicity, urbanicity, dual‐eligible status, Medicare advantage enrollment, eligibility due to end‐stage renal disease, and modified Elixhauser index.

Abbreviations: OUD, opioid use disorder; SD, standard deviation; SNF, skilled nursing facility.

**FIGURE 1:**
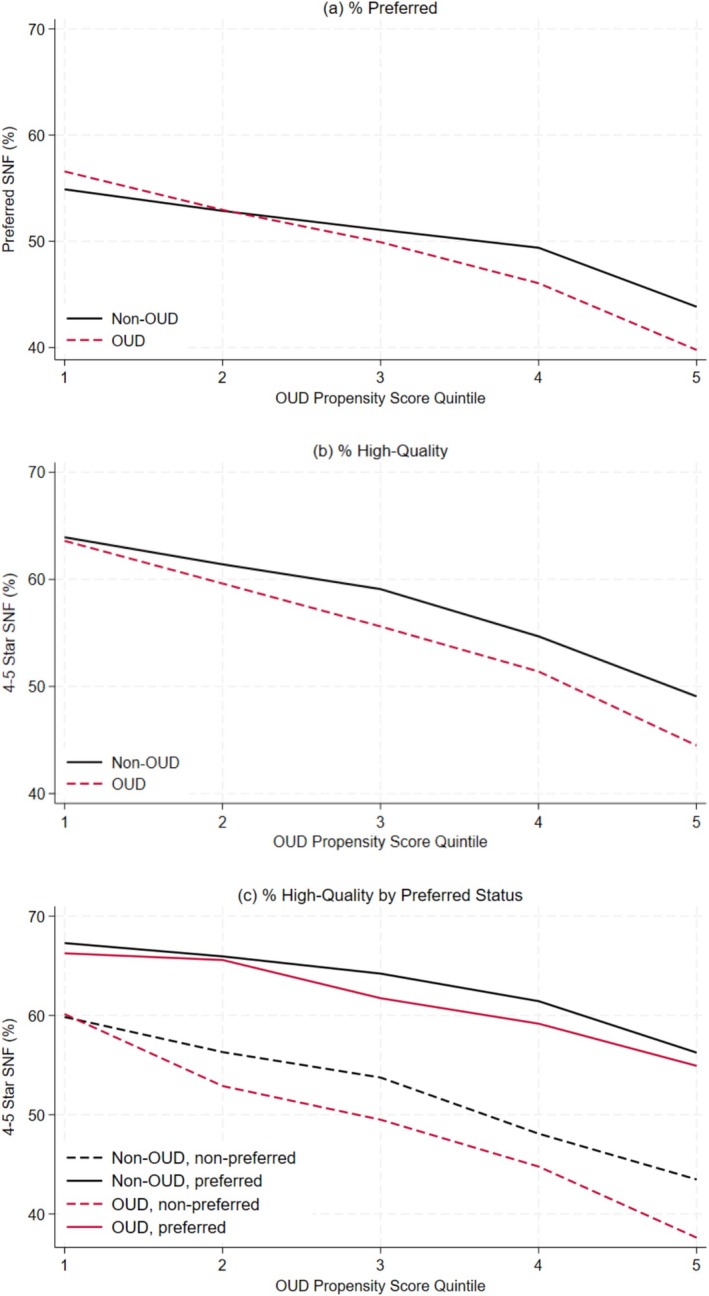
Percentage of individuals discharged to preferred and high‐quality skilled nursing facilities by propensity score quintile (*n* = 156,610). Patients with and without OUD (*n* = 156,610) were matched 1:1 on discharging hospital and date of discharge (+/−2 days). Propensity scores were estimated as a function of age, sex, race and ethnicity, urbanicity, dual‐eligible status, and modified Elixhauser index. OUD, opioid use disorder; SNF, skilled nursing facility.

Figure 1 plots the proportion of patients with and without OUD discharged to preferred and high‐quality SNFs within each propensity score quintile. Individuals with characteristics associated with a higher likelihood of having OUD (i.e., higher propensity score quintiles) were less likely to enter preferred or high‐quality SNFs regardless of whether they actually had claims‐documented OUD (Figure 1a,b). Around the second or third quintile, a disparity emerged whereby individuals with OUD were less likely than individuals without OUD to enter preferred or high‐quality SNFs, conditional on their predicted OUD risk. Figure 1c additionally shows that among individuals who entered preferred SNFs in the higher propensity score quintiles, patients with OUD were slightly less likely to be discharged to high‐quality preferred SNFs compared to those without OUD.

### 3.1 Linear Probability Models

Results from the fixed‐effect linear probability models reflect within‐hospital comparisons and are adjusted for patient‐level characteristics. In the full sample, patients with OUD were 2.5 percentage points (pp) less likely to enter a preferred SNF and 3.6 pp less likely to enter a high‐quality SNF than those without OUD (both *p* < 0.001; Table 3). Among those discharged to a preferred SNF, patients with OUD were 2.0 pp less likely to access high‐quality SNFs (*p* < 0.001; Table 3). Results were similar in the 1:1 matched sample, and in models without hospital fixed effects (Tables S3 and S4).

**TABLE 3:** Linear probability model results for the association of opioid use disorder with preferred/high‐quality skilled nursing facility status in full sample (*N* = 6,490,164).

	Preferred SNF	High‐quality SNF	High‐quality SNF, among those discharged to preferred SNF
	Coefficient [95% CI]	Coefficient [95% CI]	Coefficient [95% CI]
OUD (ref. non‐OUD)	−0.025 [−0.028, −0.022]***	−0.036 [−0.040, −0.033]***	−0.020 [−0.027, −0.013]***
Age	0.002 [0.002, 0.002]***	0.002 [0.002, 0.002]***	0.001 [0.001, 0.001]***
Female (ref. male)	0.025 [0.024, 0.026]***	0.029 [0.029, 0.030]***	0.018 [0.016, 0.019]***
Race (ref. Non‐Hispanic White)			
Unknown	0.016 [0.011, 0.021]***	0.011 [0.006, 0.015]***	0.008 [0.002, 0.013]**
Black/African American	0.018 [0.017, 0.019]***	−0.058 [−0.059, −0.057]***	−0.037 [−0.047, −0.026]***
Other	0.008 [0.003, 0.013]***	−0.012 [−0.016, −0.007]***	−0.010 [−0.017, −0.002]**
Asian/Pacific Islander	0.007 [0.004, 0.010]***	−0.001 [−0.003, 0.002]	−0.006 [−0.015, 0.002]
Hispanic	0.004 [0.002, 0.006]***	−0.026 [−0.028, −0.025]***	−0.017 [−0.023, −0.011]***
American Indian/Alaska Native	−0.013 [−0.019, −0.006]***	−0.044 [−0.049, −0.038]***	−0.020 [−0.029, −0.010]***
Dual‐eligible (ref. No)	−0.072 [−0.073, −0.071]***	−0.083 [−0.084, −0.082]***	−0.051 [−0.058, −0.043]***
Medicare advantage (ref. traditional Medicare)	−0.006 [−0.007, −0.005]***	−0.040 [−0.041, −0.039]***	−0.032 [−0.041, −0.023]***
Eligible due to ESRD (ref. No)	−0.012 [−0.014, −0.009]***	−0.027 [−0.029, −0.024]***	−0.024 [−0.033, −0.015]***
Urbanicity (ref. Metropolitan)			
Micropolitan	−0.172 [−0.174, −0.170]***	−0.015 [−0.017, −0.014]***	0.001 [−0.005, 0.008]
Small town	−0.249 [−0.251, −0.247]***	−0.014 [−0.016, −0.012]***	0.008 [0.000, 0.016]
Rural	−0.267 [−0.269, −0.264]***	0.001 [−0.001, 0.003]	0.015 [0.006, 0.024]**
Modified Elixhauser index	0.000 [0.000, 0.000]*	−0.003 [−0.003, −0.003]***	−0.002 [−0.003, −0.002]***
Sample size	6,490,164	6,490,164	3,394,386

*Note:* ***p < 0.001, **p < 0.01, *p < 0.05. Linear probability models with hospital fixed‐effects. The outcomes are preferred SNF status and high‐quality (i.e., 4–5 star) SNF. 66 singleton observations were dropped from the models in columns 1 and 2; 71 singleton observations were dropped from the model in column 3.

Abbreviations: CI, confidence interval; OUD, opioid use disorder; SNF, skilled nursing facility.

### 3.2 Choice Models

Table 4 shows marginal effects from the choice models for patients with versus without OUD. The marginal effects depict the simulated change in the probability of entering the closest SNF in one's choice set if it is preferred versus non‐preferred, or if the star rating increases by one unit. Full choice model output is available in Tables S5 and S6. Sensitivity analyses provided no evidence against the IIA assumption (Table S7).

**TABLE 4:** Marginal changes in probability of entering the closest skilled nursing facility by preferred status and quality (*n* = 156,610).

	Marginal effect of preferred status, percentage points			Marginal effect of overall quality rating (stars), percentage points		
	Non‐OUD	OUD	Difference (OUD minus non‐OUD)	Non‐OUD	OUD	Difference (OUD minus non‐OUD)
Overall choice model	12.78	11.66	−1.12*	0.71	−0.20	−0.90***
Overall choice model with IPW	12.50	11.85	−0.64	0.48	0.07	−0.42***
Quintile 1	12.81	13.03	0.23	1.14	1.21	0.08
Quintile 2	12.83	12.85	0.01	0.92	0.62	−0.31**
Quintile 3	13.05	12.90	−0.15	0.65	0.17	−0.48***
Quintile 4	12.79	11.44	−1.36	0.27	−0.22	−0.49***
Quintile 5	11.21	9.95	−1.26	−0.21	−0.84	−0.63***

*Note:* ***p < 0.001, **p < 0.01, *p < 0.05. The marginal effects depict the simulated change in the probability of going to the closest SNF in one's choice set if the facility characteristic changes by one unit. For example, the probability of entering the closest SNF when it is preferred (compared to non‐preferred) increases by 12.78 percentage points for a person without OUD and by 11.66 percentage points for a person with OUD. When the quality rating of the closest SNF increases by 1 star, the probability of entering that SNF increases by 0.71 percentage points for a person without OUD but decreases by −0.20 percentage points for a person with OUD. We re‐ran these choice models with inverse probability weights and within each quintile of the propensity score. The significance levels on the difference column refer to the significance of the interaction terms between OUD status and the facility characteristic of interest (preferred status or quality). Full choice model output is available in Tables S4 and S5. Choice sets included 11,445,400 alternatives among 156,610 individual discharges.

Abbreviations: IPW, inverse probability weights; OUD, opioid use disorder.

Preferred status increased the probability that someone without OUD entered their closest SNF by 12.8 pp, while it increased the probability for patients with OUD by 11.7 pp (difference = 1.1 pp, *p* = 0.04). This difference did not remain significant after applying IPW (*p* = 0.66) or within propensity score quintiles (Table 4), and persisted under 1:2 but not 1:3 matching ratios (Table S8). In sensitivity analyses using alternative definitions of preferred status, the differential effect of preferred status was qualitatively consistent, but only significant when “preferred” included the set of SNFs accounting for 40% or more of the hospital's discharges (Table S9).

When the quality rating of the closest SNF increased by one star, the probability of entering that SNF increased by 0.7 pp for a person without OUD, but decreased by 0.2 pp for a person with OUD (difference = 0.9 pp, *p* < 0.001). This difference remained significant after IPW (*p* < 0.001), and within all but the lowest propensity score quintile (Table 4). It was robust to using 1:2 or 1:3 matching ratios (Table S8).

## 4 Discussion

Using national Medicare data, we found that patients with OUD were less likely to access high‐quality SNFs relative to a matched sample without OUD, even when holding choice sets fixed and accounting for other SNF attributes. Patients with OUD were also slightly less likely to access preferred SNFs overall. However, in choice models, the differential effect of preferred status was small and was not robust to adjustment for patient‐level confounding, nor to stricter definitions of “preferred” status. These results suggest limited evidence of systematic exclusion of patients with OUD from preferred SNFs. Among patients who entered preferred SNFs, patients with OUD still entered lower‐quality preferred SNFs. Thus, high‐volume referral linkages, even if accessed equitably, do not seem to entirely mitigate the quality‐related differences in SNF placement.

Several mechanisms could explain differential access to high‐quality SNFs for people with OUD. Higher‐rated SNFs may be more selective in their admissions in efforts to maintain their higher ratings [33, 36, 47, 48] or because they have more consistent referral flows in general, even if not from one specific hospital [35, 37, 38]. Additionally, hospital discharge planners may prioritize other factors over star ratings when placing patients with OUD, such as the facility's likelihood of accepting the patient or the presence of specialized behavioral health services, as analyses of the SNF industry suggest that some hospitals include SNFs with lower star ratings in their preferred networks for these reasons [33]. These facilities may specialize in caring for people with OUD in ways we could not measure. Therefore, we cannot unequivocally infer from this study that discharge to lower‐quality SNFs negatively impacts outcomes of patients with OUD. Future research should examine how SNF quality metrics and case‐mix specifically impact outcomes of patients with OUD and whether quality measures that are more directly tied to OUD‐related care can better capture care quality for this population. Our study further found that very few SNFs admitted large shares of patients with OUD. These small percentages could limit the incentives for SNFs to invest in developing OUD‐related care capacities, despite growing demands for SNF care among this population. There is a clinical and legal imperative for OUD‐related care (e.g., medication for OUD) to become more accessible across SNF settings, not just in a few specialized facilities. Coordination between SNFs and community‐based providers such as opioid treatment programs and hospital addiction consult services could help address the OUD care gap in SNFs [4, 19].

Discharge to preferred and high‐quality SNFs decreased and OUD‐related disparities widened as patients' demographic and clinical profiles (summarily identified by propensity scores) became more associated with OUD. This finding is consistent with qualitative research in which SNF administrators cited factors such as younger age and Medicaid's lower reimbursement rates—which, in our sample, are both predictive of OUD—as reasons for not admitting patients with OUD [4]. Additionally, dual‐eligibility and race and ethnicity have been independently linked to disparities accessing high‐quality post‐acute care [43, 44, 49] and found to compound OUD‐related barriers to accessing SNFs [4, 50]. Although OUD involves specialized treatment needs that may create unique barriers to accessing SNFs, these patterns of intersectionality highlight the importance of policies that address overlapping barriers to SNF access, such as addressing financial disincentives driven by low Medicaid reimbursement rates [5].

### 4.1 Limitations

Due to the nature of claims data, we could only observe SNF admissions, not referrals. A Massachusetts study found that over one‐third of patients with OUD who were referred to SNFs were rejected and ultimately never placed in any SNF [8]. This could introduce selection bias in our sample. We expect this would lead to underestimation of disparities in SNF quality because individuals never admitted to SNFs may have faced the greatest barriers to placement, suggesting they would have been more likely to enter lower‐quality SNFs had they been admitted.

We also could not observe whether disparities stemmed from patient preferences, SNF selectivity, hospital steering, and/or clinically appropriate matching based on specialized care needs. We lacked measures of OUD severity and treatment needs (e.g., medications for OUD, antibiotic therapy, or wound care), and of facilities' capacity to provide such services, which could confound the relationship between facility attributes and the placement of patients with OUD, even after matching on observed patient characteristics and holding multiple SNF characteristics constant in our simulations.

We were also unable to observe the formal “preferred” relationships between hospitals and SNFs because there is no standardized measure of preferred networks at a national level. Instead, we proxied for these relationships using shared discharge volumes. This approach reflects informal relationships based on referral patterns used in practice, rather than formal preferred SNF designations and the explicit investments associated with those designations.

## 5 Conclusion

Medicare beneficiaries with OUD face disparities accessing high‐quality SNFs. Findings regarding access to hospitals' preferred SNFs were less consistent but suggested that preferred networks alone may not be sufficient to eliminate quality‐related disparities. Policy levers can include reimbursement and regulatory models that foster equitable SNF admission practices, as well as support for high‐quality SNFs in developing capacity to manage OUD care.

## Funding

This work was funded by the National Institutes of Health under grant R21DA053518 from the National Institute on Drug Abuse (NIDA). Talia S. Benheim received support from AHRQ grant T32HS000011. Simeon D. Kimmel received support from NIDA grant 5K23DA054363 and a Boston University Department of Medicine Career Investment Award.

## Conflicts of Interest

Patience Moyo Dow reports grant funding from the National Institutes of Health and the Commonwealth Fund. Simeon D. Kimmel reports consulting fees from the Massachusetts Department of Public Health's Bureau of Substance Addiction Services paid to his institution. The other authors declare no conflicts of interest.

## Supporting information


**Figure S1:** Cohort selection flowchart.
**Figure S2:** Unweighted and weighted propensity score distributions after trimming.
**Figure S3:** Cumulative distribution function of SNF‐level OUD volumes (among eligible hospital‐to‐SNF transfers).
**Table S1:** ICD‐10‐CM and ICD‐10‐PCS diagnosis codes for opioid use disorder, opioid dependence, and opioid use disorder treatment.
**Table S2:** Baseline characteristics of SNFs by SNF‐level OUD volumes (among eligible hospital‐to‐SNF transfers).
**Table S3:** Linear probability model results for the association of opioid use disorder with preferred/high‐quality skilled nursing facility status in the matched subsample (*n* = 156,610).
**Table S4:** Linear probability model results for the association of opioid use disorder with preferred/high‐quality skilled nursing facility status in the full sample, without hospital fixed effects (*N* = 6,490,230).
**Table S5:** Choice model results (*n* = 156,610 discharges).
**Table S6:** Choice model results with inverse probability weighting (*n* = 156,610 discharges).
**Table S7:** Independence from irrelevant alternatives assumption.
**Table S8:** Robustness of choice model results to alternative matching ratios (1:2 and 1:3).
**Table S9:** Marginal changes in probability of entering the closest skilled nursing facility by varying definitions of preferred status, with and without inverse probability weighting.

## Data Availability

The data that support the findings of this study are available from the Centers for Medicare & Medicaid Services (CMS). Restrictions apply to the availability of these data, which were used under license for this study. Data are available from https://resdac.org/ with the permission of CMS.
