# Supplementary material for: Post‐Hospital Access to Preferred and High‐Quality Skilled Nursing Facilities for Patients With Opioid Use Disorder
Source: Health Serv Res. 2026 Mar 31;61(2):e70110. doi: 10.1111/1475-6773.70110 (PMC13077784; doi:10.1111/1475-6773.70110)
Supplement: Supplementary file 1 — Figure S1: Cohort selection flowchart. Figure S2: Unweighted and weighted propensity score distributions after trimming. Figure S3: Cumulative distribution function of SNF‐level OUD volumes (among eligible hospital‐to‐SNF transfers). Table S1: ICD‐10‐CM and ICD‐10‐PCS diagnosis codes for opioid use disorder, opioid dependence, and opioid use disorder treatment. Table S2: Baseline characteristics of SNFs by SNF‐level OUD volumes (among eligible hospital‐to‐SNF transfers). Table S3: Linear probability model results for the association of opioid use disorder with preferred/high‐quality skilled nursing facility status in the matched subsample (n = 156,610). Table S4: Linear probability model results for the association of opioid use disorder with preferred/high‐quality skilled nursing facility status in the full sample, without hospital fixed effects (N = 6,490,230). Table S5: Choice model results (n = 156,610 discharges). Table S6: Choice model results with inverse probability weighting (n = 156,610 discharges). Table S7: Independence from irrelevant alternatives assumption. Table S8: Robustness of choice model results to alternative matching ratios (1:2 and 1:3). Table S9: Marginal changes in probability of entering the closest skilled nursing facility by varying definitions of preferred status, with and without inverse probability weighting. [file HESR-61-e70110-s001.docx]

**Supplemental Appendix**

[**eFigure 1.** Cohort selection flowchart 2](#_Toc217312549)

[**eTable 1.** ICD-10-CM and ICD-10-PCS diagnosis codes for opioid use disorder, opioid dependence, and opioid use disorder treatment 3](#_Toc217312550)

[**eFigure 2.** Unweighted and weighted propensity score distributions after trimming 4](#_Toc217312551)

[**eFigure 3.** Cumulative distribution function of SNF-level OUD volumes (among eligible hospital-to-SNF transfers) 5](#_Toc217312552)

[**eTable 2.** Baseline characteristics of SNFs by SNF-level OUD volumes (among eligible hospital-to-SNF transfers) 6](#_Toc217312553)

[**eTable 3.** Linear probability model results for the association of opioid use disorder with preferred/high-quality skilled nursing facility status in the matched subsample (n=156,610) 7](#_Toc217312554)

[**eTable 4.** Linear probability model results for the association of opioid use disorder with preferred/high-quality skilled nursing facility status in the full sample, without hospital fixed effects (N=6,490,230) 8](#_Toc217312555)

[**eTable 5.** Choice model results (n=156,610 discharges) 9](#_Toc217312556)

[**eTable 6.** Choice model results with inverse probability weighting (n=156,610 discharges) 10](#_Toc217312557)

[**eTable 7.** Independence from irrelevant alternatives assumption. 11](#_Toc217312558)

[**eTable 8.** Robustness of choice model results to alternative matching ratios (1:2 and 1:3) 12](#_Toc217312559)

[**eTable 9.** Marginal changes in probability of entering the closest skilled nursing facility by varying definitions of preferred status, with and without inverse probability weighting 13](#_Toc217312560)

# **eFigure 1.** Cohort selection flowchart


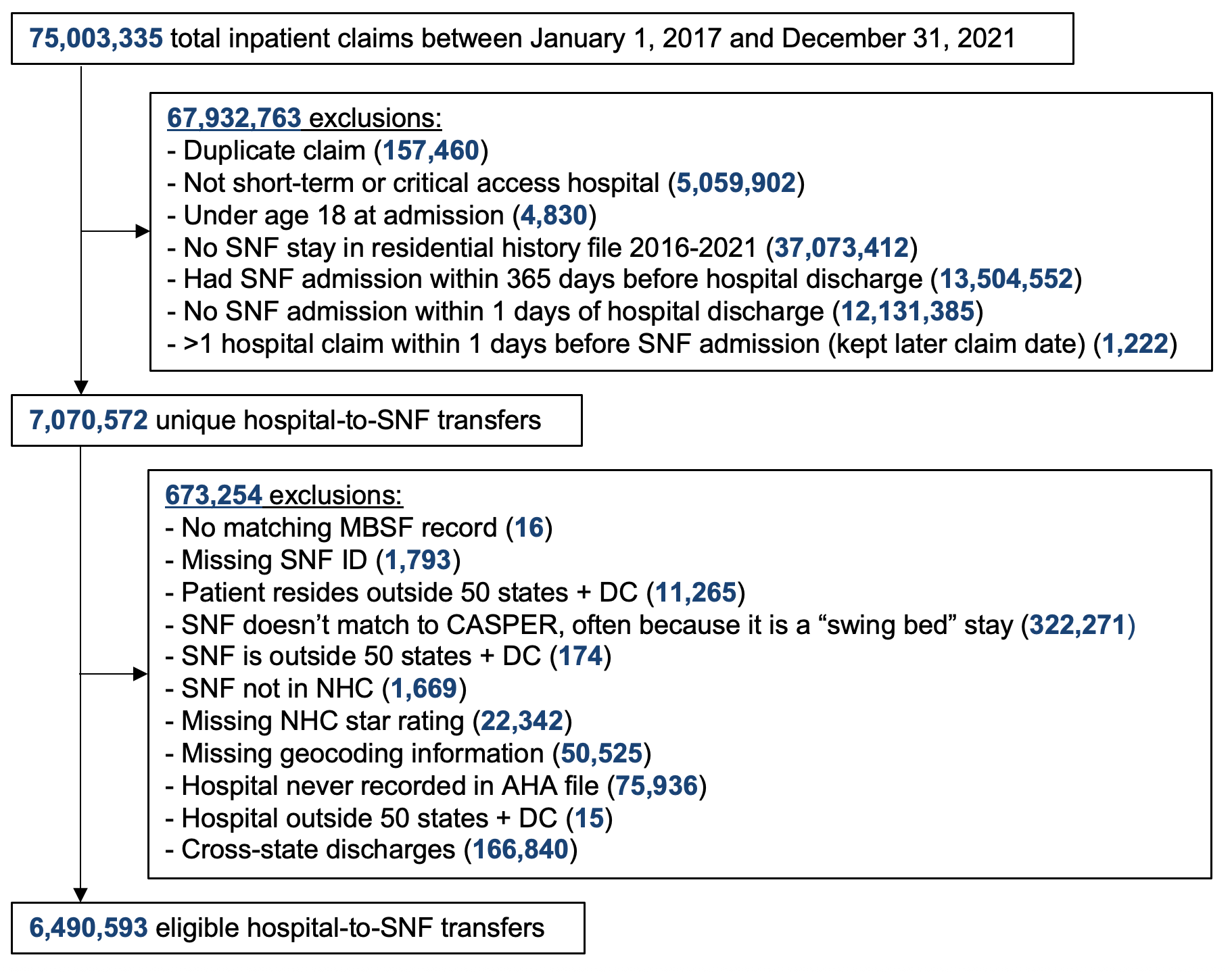


# **eTable 1.** ICD-10-CM and ICD-10-PCS diagnosis codes for opioid use disorder, opioid dependence, and opioid use disorder treatment

| **ICD-10-CM:** F11.10, F11.120, F11.121, F11.122, F11.129, F11.14, F11.150, F11.151, F11.159, F11.181, F11.182, F11.188, F11.19, F11.20, F11.220, F11.221, F11.222, F11.229, F11.23, F11.24, F11.250, F11.251, F11.259, F11.281, F11.282, F11.288, F11.29, F11.90, F11.920, F11.921, F11.922, F11.929, F11.93, F11.94, F11.950, F11.951, F11.959, F11.981, F11.982, F11.988, F11.99, T40.0X1A, T40.0X2A, T40.0X3A, T40.0X4A, T40.1X1A, T40.1X2A, T40.1X3A, T40.1X4A, T40.2X1A, T40.2X2A, T40.2X3A, T40.2X4A, T40.3X1A, T40.3X2A, T40.3X3A, T40.3X4A, T40.3X5A, T40.4X1A, T40.4X2A, T40.4X3A, T40.4X4A, T40.411A, T40.412A, T40.413A, T40.414A, T40.415A, T40.421A, T40.422A, T40.423A, T40.424A, T40.425A, T40.491A, T40.492A, T40.493A, T40.494A, T40.495A, T40.601A, T40.602A, T40.603A, T40.604A, T40.691A, T40.692A, T40.693A, T40.694A  **ICD-10-PCS:** HZ81ZZZ, HZ84ZZZ, HZ85ZZZ, HZ86ZZZ, HZ91ZZZ, HZ94ZZZ, HZ95ZZZ, HZ96ZZZ |
| --- |

# **eFigure 2.** Unweighted and weighted propensity score distributions after trimming


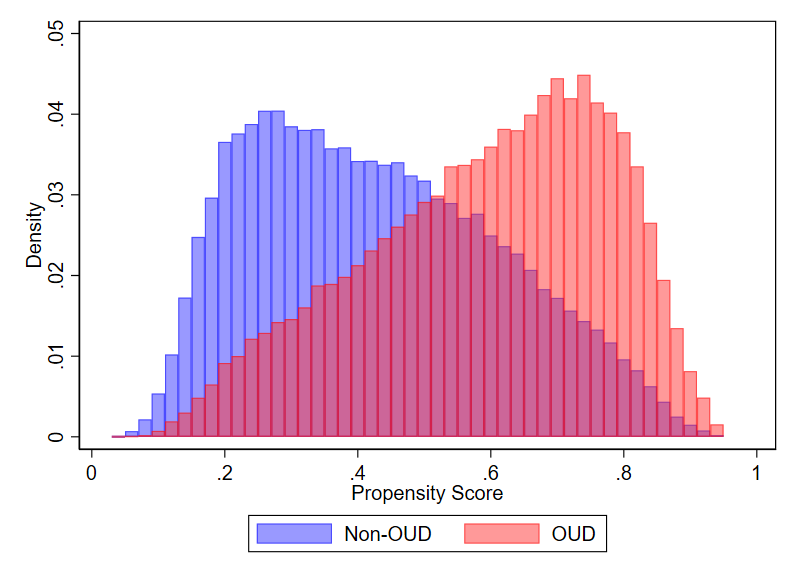

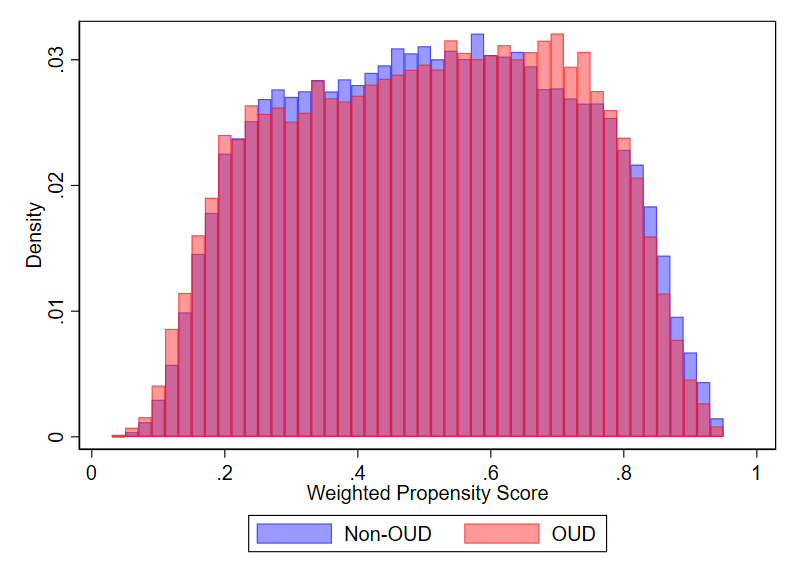


First, we matched people with and without OUD 1:1 on the discharging hospital and date (+/- 2 days). There was a 97.4% match rate (n=156,646). Within the matched sample, we generated propensity scores using a logit model with OUD as the outcome and the following predictors: age (as a two-piece linear spline with the knot set at 65), dual-eligible status, Medicare Advantage enrollment, eligibility due to end-stage renal disease, recorded sex, recorded race and ethnicity, urbanicity, and modified Elixhauser index. We excluded 36 observations that lacked common support (people with OUD whose propensity scores were outside the range of those without OUD and vice versa). After trimming, our matched sample had 156,610 observations (78,318 without OUD and 78,292 with OUD).

# **eFigure 3.** Cumulative distribution function of SNF-level OUD volumes (among eligible hospital-to-SNF transfers)


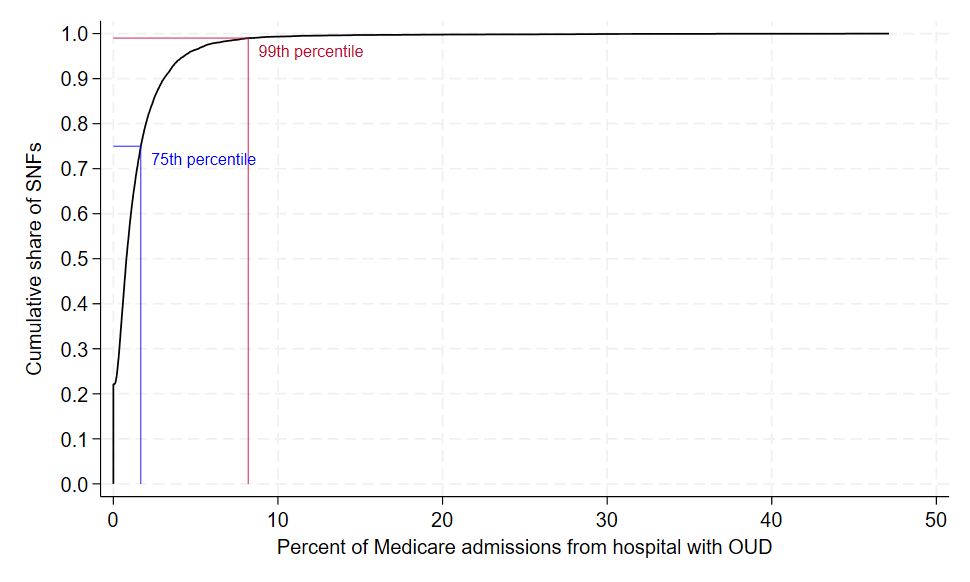


For each SNF, we calculated the proportion of Medicare inpatient discharges from our analytic sample (N=6,490,593) that had an OUD diagnosis over the course of the study period. To ensure reliable denominators for this analysis, we excluded SNFs with less than 25 total admissions from our sample over the study period. This figure shows the cumulative distribution function of these SNF-level OUD proportions.

For example, 22% of SNFs had no admissions from our analytic sample with OUD. In the median SNF, 0.8% of admissions had OUD; in 75% of SNFs, less than 1.7% of admissions had OUD; in 95%, less than 4.3% of admissions had OUD; and in 99%, less than 8.1% of admissions had OUD. Though patients with other insurance types and those admitted from the community are not included in this analysis, these results suggest that only a small proportion of SNFs serve as “high-volume” providers of care specifically for Medicare beneficiaries with OUD.

# **eTable 2.** Baseline characteristics of SNFs by SNF-level OUD volumes (among eligible hospital-to-SNF transfers)

|  | **No OUD cases** | **>0 to ≤1% OUD share** | **>1 to ≤5% OUD share** | **>5% OUD share** |
| --- | --- | --- | --- | --- |
|  | n=3,153 | n=5,088 | n=5,496 | n=505 |
| Ownership |  |  |  |  |
| For-profit | 62.9% | 65.9% | 80.9% | 87.9% |
| Non-profit | 29.0% | 28.9% | 15.4% | 7.1% |
| Government | 8.1% | 5.2% | 3.8% | 5.0% |
| Multifacility chain | 52.5% | 59.1% | 65.1% | 61.8% |
| Hospital-affiliated | 4.0% | 4.7% | 2.7% | 1.8% |
| % Occupancy rate, Mean (SD) | 77.1 (17.8) | 80.9 (17.0) | 79.0 (17.1) | 78.9 (17.3) |
| Bed Size, Mean (SD) | 89.3 (50.2) | 121.3 (66.0) | 115.2 (58.4) | 121.9 (78.7) |
| % Medicaid, Mean (SD) | 58.7 (24.3) | 50.7 (25.4) | 60.6 (22.9) | 70.5 (21.0) |
| Overall quality star rating, Mean (SD) | 3.22 (1.41) | 3.40 (1.38) | 3.02 (1.40) | 2.72 (1.39) |

Analysis is restricted to 14,242 SNFs with at least 25 eligible admissions from our analytic sample over the course of the study period. SNFs are grouped by share of admissions with an OUD diagnosis over the study period.

Abbreviations: OUD, opioid use disorder; SNF, skilled nursing facility.

# **eTable 3.** Linear probability model results for the association of opioid use disorder with preferred/high-quality skilled nursing facility status in the matched subsample (n=156,610)

|  | **Preferred SNF**  Coefficient [95% CI] | **High-Quality SNF**  Coefficient [95% CI] |
| --- | --- | --- |
| OUD (ref. non-OUD) | -0.013 [-0.018, -0.008] *** | -0.028 [-0.033, -0.023] *** |
| Age | 0.003 [0.003, 0.003] *** | 0.003 [0.003, 0.003] *** |
| Female (ref. Male) | 0.038 [0.033, 0.043] *** | 0.037 [0.032, 0.041] *** |
| Race (ref. Non-Hispanic White) |  |  |
| Unknown | 0.037 [0.005, 0.069] * | 0.020 [-0.009, 0.049] |
| Black/African American | 0.017 [0.008, 0.026] *** | -0.043 [-0.052, -0.035] *** |
| Other | -0.002 [-0.035, 0.032] | -0.020 [-0.051, 0.010] |
| Asian/Pacific Islander | -0.016 [-0.038, 0.006] | 0.008 [-0.012, 0.028] |
| Hispanic | 0.000 [-0.011, 0.011] | -0.023 [-0.034, -0.013] *** |
| American Indian/Alaska Native | -0.007 [-0.038, 0.024] | -0.047 [-0.077, -0.017] ** |
| Dual-eligible (ref. No) | -0.076 [-0.082, -0.070] *** | -0.085 [-0.091, -0.080] *** |
| Medicare Advantage (ref. No) | -0.016 [-0.021, -0.010] *** | -0.043 [-0.048, -0.038] *** |
| Eligible due to ESRD (ref. No) | 0.019 [0.003, 0.034] * | -0.005 [-0.020, 0.010] |
| Urbanicity (ref. Metropolitan) |  |  |
| Micropolitan | -0.137 [-0.149, -0.126] *** | -0.004 [-0.015, 0.007] |
| Small town | -0.228 [-0.243, -0.213] *** | -0.018 [-0.033, -0.003] * |
| Rural | -0.228 [-0.245, -0.210] *** | -0.003 [-0.020, 0.014] |
| Modified Elixhauser index | 0.001 [0.000, 0.002] * | -0.002 [-0.003, -0.001] *** |

*** p<0.001, ** p<0.01, * p<0.05. Linear probability models with hospital fixed-effects. The outcomes are preferred SNF status and high-quality (i.e., 4-5 star) SNF.

Abbreviations: OUD, opioid use disorder; SNF, skilled nursing facility; ESRD, end-stage renal disease.

# **eTable 4.** Linear probability model results for the association of opioid use disorder with preferred/high-quality skilled nursing facility status in the full sample, without hospital fixed effects (N=6,490,230)

|  | **Preferred SNF**  Coefficient [95% CI] | **High-Quality SNF**  Coefficient [95% CI] |
| --- | --- | --- |
| OUD (ref. non-OUD) | -0.025 [-0.028, -0.021] *** | -0.019 [-0.022, -0.015] *** |
| Age | 0.002 [0.002, 0.002] *** | 0.003 [0.003, 0.003] *** |
| Female (ref. Male) | 0.026 [0.026, 0.027] *** | 0.029 [0.028, 0.030] *** |
| Race (ref. Non-Hispanic White) |  |  |
| Unknown | 0.006 [0.001, 0.011] * | 0.037 [0.032, 0.042] *** |
| Black/African American | 0.004 [0.003, 0.005] *** | -0.093 [-0.095, -0.092] *** |
| Other | 0.002 [-0.002, 0.007] | 0.034 [0.030, 0.039] *** |
| Asian/Pacific Islander | -0.006 [-0.009, -0.003] *** | 0.070 [0.068, 0.073] *** |
| Hispanic | -0.005 [-0.007, -0.004] *** | -0.025 [-0.027, -0.024] *** |
| American Indian/Alaska Native | -0.001 [-0.007, 0.005] | -0.045 [-0.051, -0.039] *** |
| Dual-eligible (ref. No) | -0.065 [-0.066, -0.064] *** | -0.095 [-0.096, -0.094] *** |
| Medicare Advantage (ref. No) | -0.012 [-0.013, -0.011] *** | -0.043 [-0.044, -0.042] *** |
| Eligible due to ESRD (ref. No) | -0.014 [-0.017, -0.012] *** | -0.030 [-0.033, -0.028] *** |
| Urbanicity (ref. Metropolitan) |  |  |
| Micropolitan | -0.032 [-0.034, -0.031] *** | -0.091 [-0.093, -0.090] *** |
| Small town | -0.121 [-0.123, -0.119] *** | -0.079 [-0.081, -0.077] *** |
| Rural | -0.134 [-0.137, -0.132] *** | -0.047 [-0.050, -0.045] *** |
| Modified Elixhauser index | -0.001 [-0.001, -0.001] *** | -0.003 [-0.003, -0.003] *** |

*** p<0.001, ** p<0.01, * p<0.05. Linear probability models. The outcomes are preferred SNF status and high-quality (i.e., 4-5 star) SNF.

Abbreviations: OUD, opioid use disorder; SNF, skilled nursing facility; ESRD, end-stage renal disease.

# **eTable 5.** Choice model results (n=156,610 discharges)

|  | **Coefficient** | **Std. Err.** | **95% CI** |
| --- | --- | --- | --- |
| Preferred SNF (ref. Non-preferred) | 1.390 | 0.069 | 1.256, 1.525*** |
| Preferred SNF * OUD | -0.056 | 0.028 | -0.110, -0.001* |
| Overall quality star rating | 0.086 | 0.005 | 0.075, 0.097*** |
| Overall quality star rating * OUD | -0.113 | 0.016 | -0.144, -0.082*** |
| Home-SNF Distance | -0.059 | 0.003 | -0.065, -0.053*** |
| Home-SNF Distance * OUD | 0.010 | 0.003 | 0.004, 0.016** |
| Hospital-SNF Distance | -0.034 | 0.002 | -0.037, -0.030*** |
| Hospital-SNF Distance * OUD | 0.007 | 0.003 | 0.002, 0.012** |
| Ownership (ref. For-profit) |  |  |  |
| Non-profit | -0.112 | 0.024 | -0.159, -0.066*** |
| Government | -0.193 | 0.087 | -0.364, -0.021* |
| Ownership * OUD |  |  |  |
| Non-profit | -0.244 | 0.038 | -0.318, -0.171*** |
| Government | -0.140 | 0.045 | -0.228, -0.051** |
| Multifacility chain (ref. No) | 0.106 | 0.015 | 0.077, 0.135*** |
| Multifacility chain * OUD | 0.062 | 0.016 | 0.031, 0.092*** |
| Occupancy rate | 0.003 | 0.001 | 0.001, 0.005** |
| Occupancy rate * OUD | 0.000 | 0.001 | -0.001, 0.001 |
| Bed size | 0.002 | 0.000 | 0.002, 0.003*** |
| Bed size * OUD | 0.000 | 0.000 | 0.000, 0.000 |
| Hospital-affiliated (ref. No) | 0.127 | 0.043 | 0.042, 0.212** |
| Hospital-affiliated * OUD | 0.207 | 0.029 | 0.151, 0.263*** |
| % Medicaid | -0.009 | 0.001 | -0.010, -0.008*** |
| % Medicaid * OUD | 0.004 | 0.000 | 0.003, 0.005*** |

*** p<0.001, ** p<0.01, * p<0.05. Conditional logistic regression with individual choice set fixed effects. Standard errors are clustered at the state level. Choice sets included 11,445,400 alternatives among 156,610 individual discharges.

Abbreviations: OUD, opioid use disorder; SNF, skilled nursing facility.

# **eTable 6.** Choice model results with inverse probability weighting (n=156,610 discharges)

|  | **Coefficient** | **Std. Err.** | **95% CI** |
| --- | --- | --- | --- |
| Preferred SNF (ref. Non-preferred) | 1.362 | 0.069 | 1.226, 1.497*** |
| Preferred SNF * OUD | -0.012 | 0.027 | -0.064, 0.040 |
| Overall quality star rating | 0.060 | 0.006 | 0.049, 0.071*** |
| Overall quality star rating * OUD | -0.051 | 0.013 | -0.077, -0.025*** |
| Home-SNF Distance | -0.057 | 0.003 | -0.063, -0.051*** |
| Home-SNF Distance * OUD | 0.006 | 0.003 | -0.001, 0.012 |
| Hospital-SNF Distance | -0.032 | 0.002 | -0.035, -0.029*** |
| Hospital-SNF Distance * OUD | 0.002 | 0.003 | -0.003, 0.008 |
| Ownership (ref. For-profit) |  |  |  |
| Non-profit | -0.196 | 0.029 | -0.252, -0.140*** |
| Government | -0.234 | 0.094 | -0.419, -0.049* |
| Ownership * OUD |  |  |  |
| Non-profit | -0.067 | 0.020 | -0.106, -0.027** |
| Government | -0.055 | 0.024 | -0.102, -0.008* |
| Multifacility chain (ref. No) | 0.124 | 0.015 | 0.095, 0.153*** |
| Multifacility chain * OUD | 0.020 | 0.018 | -0.015, 0.055 |
| Occupancy rate | 0.003 | 0.001 | 0.001, 0.005** |
| Occupancy rate * OUD | 0.001 | 0.001 | -0.001, 0.002 |
| Bed size | 0.002 | 0.000 | 0.002, 0.003*** |
| Bed size * OUD | 0.000 | 0.000 | 0.000, 0.000 |
| Hospital-affiliated (ref. No) | 0.232 | 0.048 | 0.137, 0.326*** |
| Hospital-affiliated * OUD | 0.003 | 0.028 | -0.052, 0.058 |
| % Medicaid | -0.007 | 0.001 | -0.009, -0.006*** |
| % Medicaid * OUD | 0.001 | 0.000 | 0.000, 0.002* |

*** p<0.001, ** p<0.01, * p<0.05. Conditional logistic regression with individual choice set fixed effects. Standard errors are clustered at the state level. Choice sets included 11,445,400 alternatives among 156,610 individual discharges.

Abbreviations: OUD, opioid use disorder; SNF, skilled nursing facility.

# **eTable 7.** Independence from irrelevant alternatives assumption.

The conditional logit model requires the assumption of independence from irrelevant alternatives (IIA). This assumption means that the relative probability of choosing one option over another should not be affected by the presence of other, unrelated options. While we cannot directly test IIA, we randomly dropped 5%, 10%, and 20% of unchosen SNFs from each person’s choice set and re-estimated the model to examine whether coefficients remained stable when unrelated. We found that the parameter estimates remained similar in both magnitude and direction after omitting random options.

|  | **Full** | **5% omitted** | **10% omitted** | **20% omitted** |
| --- | --- | --- | --- | --- |
| Preferred | 1.390 | 1.392 | 1.393 | 1.399 |
| Preferred * OUD | -0.056 | -0.055 | -0.054 | -0.056 |
| Overall star rating | 0.086 | 0.086 | 0.086 | 0.087 |
| Overall star rating * OUD | -0.113 | -0.113 | -0.112 | -0.115 |
| Home-SNF distance | -0.059 | -0.059 | -0.059 | -0.059 |
| Home-SNF distance * OUD | 0.010 | 0.010 | 0.010 | 0.010 |
| Hospital-SNF distance | -0.034 | -0.033 | -0.033 | -0.033 |
| Hospital-SNF distance * OUD | 0.007 | 0.007 | 0.007 | 0.007 |
| Occupancy rate | 0.003 | 0.003 | 0.003 | 0.003 |
| Occupancy rate * OUD | 0.000 | 0.000 | 0.000 | 0.000 |
| % Medicaid | -0.009 | -0.009 | -0.009 | -0.009 |
| % Medicaid * OUD | 0.004 | 0.004 | 0.004 | 0.004 |
| Multifacility chain | 0.106 | 0.105 | 0.107 | 0.108 |
| Multifacility chain * OUD | 0.062 | 0.062 | 0.063 | 0.062 |
| Bed size | 0.002 | 0.002 | 0.002 | 0.002 |
| Bed size * OUD | 0.000 | 0.000 | 0.000 | 0.000 |
| Non-profit | -0.112 | -0.114 | -0.113 | -0.115 |
| Non-profit * OUD | -0.244 | -0.244 | -0.246 | -0.243 |
| Government | -0.193 | -0.197 | -0.194 | -0.198 |
| Government * OUD | -0.140 | -0.137 | -0.144 | -0.143 |
| Hospital-affiliated | 0.127 | 0.127 | 0.125 | 0.116 |
| Hospital-affiliated * OUD | 0.207 | 0.206 | 0.208 | 0.219 |

Abbreviations: OUD, opioid use disorder; SNF, skilled nursing facility.

# **eTable 8.** Robustness of choice model results to alternative matching ratios (1:2 and 1:3)

Marginal changes in probability of entering the closest skilled nursing facility, with and without inverse probability weighting, in a 1:2 matched sample of patients with OUD to patients without OUD (n=231,460). In this analysis, each individual with OUD was matched with up to two individuals without OUD by discharging hospital and date (+/- 2 days). 97.2% of observations had at least 1 match. 93.3% had 2 matches.

|  | **Marginal effect of preferred status,**  **percentage points** | | | **Marginal effect of overall quality rating (stars),**  **percentage points** | | |
| --- | --- | --- | --- | --- | --- | --- |
|  | Non-OUD | OUD | Difference  (OUD minus Non-OUD) | Non-OUD | OUD | Difference  (OUD minus Non-OUD) |
| Overall choice model | 12.76 | 11.65 | -1.11* | 0.71 | -0.20 | -0.91*** |
| Overall choice model with IPW | 12.63 | 11.91 | -0.73 | 0.56 | 0.16 | -0.39*** |

*** p<0.001, ** p<0.01, * p<0.05. The marginal effects depict the simulated change in the probability of going to the closest SNF in one’s choice set if the facility characteristic changes by one unit.

Abbreviations: IPW, inverse probability weights; OUD, opioid use disorder.

Marginal changes in probability of entering the closest skilled nursing facility, with and without inverse probability weighting, in a 1:3 matched sample of patients with OUD to patients without OUD (n=302,049). In this analysis, each individual with OUD was matched with up to three individuals without OUD by discharging hospital and date (+/- 2 days). 97.1% of observations had at least 1 match. 88.4% had 3 matches.

|  | **Marginal effect of preferred status,**  **percentage points** | | | **Marginal effect of overall quality rating (stars),**  **percentage points** | | |
| --- | --- | --- | --- | --- | --- | --- |
|  | Non-OUD | OUD | Difference  (OUD minus Non-OUD) | Non-OUD | OUD | Difference  (OUD minus Non-OUD) |
| Overall choice model | 12.59 | 11.65 | -0.93 | 0.71 | -0.20 | -0.91*** |
| Overall choice model with IPW | 12.47 | 11.96 | -0.51 | 0.59 | 0.20 | -0.39*** |

*** p<0.001, ** p<0.01, * p<0.05. The marginal effects depict the simulated change in the probability of going to the closest SNF in one’s choice set if the facility characteristic changes by one unit.

Abbreviations: IPW, inverse probability weights; OUD, opioid use disorder.

# **eTable 9.** Marginal changes in probability of entering the closest skilled nursing facility by varying definitions of preferred status, with and without inverse probability weighting

|  | **Marginal effect of preferred status,**  **percentage points** | | | **Marginal effect of preferred status with inverse probability weighting,**  **percentage points** | | |
| --- | --- | --- | --- | --- | --- | --- |
|  | Non-OUD | OUD | Difference  (OUD minus Non-OUD) | Non-OUD | OUD | Difference  (OUD minus Non-OUD) |
| Preferred = cumulative 10% | 12.06 | 11.76 | -0.30 | 12.05 | 11.56 | -0.50 |
| Preferred = cumulative 20% | 12.23 | 11.69 | -0.54 | 12.14 | 11.57 | -0.57 |
| Preferred = cumulative 30% | 12.50 | 11.57 | -0.93 | 12.31 | 11.57 | -0.74 |
| Preferred = cumulative 40% | 12.79 | 11.61 | -1.18* | 12.56 | 11.73 | -0.82 |
| Preferred = cumulative 50% | 12.78 | 11.66 | -1.12* | 12.50 | 11.85 | -0.64 |
| Preferred = cumulative 60% | 12.78 | 11.55 | -1.23* | 12.45 | 11.77 | -0.68 |
| Preferred = cumulative 70% | 12.53 | 11.21 | -1.32** | 12.18 | 11.48 | -0.70 |
| Preferred = cumulative 80% | 11.96 | 10.58 | -1.38*** | 11.63 | 10.91 | -0.71 |

*** p<0.001, ** p<0.01, * p<0.05. The marginal effects depict the simulated change in the probability of going to the closest SNF in one’s choice set if the SNF is preferred versus non-preferred. The significance levels on the difference columns refer to the significance of the interaction terms between OUD status and preferred status.

Abbreviations: OUD, opioid use disorder; SNF, skilled nursing facility.
